# An unusual case of small bowel obstruction because of body packing

**DOI:** 10.1093/jscr/rjad207

**Published:** 2023-04-25

**Authors:** Alexandra L Boucher, Nikunj Shah, Daniel H Brynien, Jason M Bregg, Shinichiro Yokota

**Affiliations:** College of Medicine, Drexel University, Philadelphia, PA, USA; Lake Erie College of Osteopathic Medicine, Erie, PA, USA; Department of Surgery, Allegheny Health Network, Pittsburgh, PA, USA; Department of Surgery, Allegheny Health Network, Pittsburgh, PA, USA; Department of Surgery, Allegheny Health Network, Pittsburgh, PA, USA

## Abstract

Body packing is the internal concealment of illicit drugs for the purpose of transportation and evasion of law enforcement. It is associated with medical complications such as acute toxicity from ingested drug, bowel obstruction or perforation. It is rare to require surgical intervention with a small amount of ingested drug packet. Here, we present the case of a young adult male who presented with abdominal pain for 3 days. The patient admitted ingesting a condom filled with suboxone several years ago and denied recent ingestion. The patient was found to have small bowel obstruction with ingested foreign body being a transition point on CT scan, requiring exploratory laparotomy and small bowel resection.

## INTRODUCTION

Body packing is a way to transport illicit drugs by internal concealment [1]. Some deliver drugs across international borders hidden in anatomical cavities such as the mouth, rectum, intestine and vagina [2, 3]. It is also used to introduce illicit drugs into prisons or to conceal these materials from police search [3]. Small bowel obstruction is an infrequent complication associated with body packing and there are limited reports in the literature [4]. We present a case of a male patient with a remote history of body packing who developed a mechanical small bowel obstruction because of suboxone film encased in a condom requiring exploratory laparotomy.

## CASE REPORT

A previously healthy young male with no significant past medical or surgical history presented to the emergency department with abdominal pain. The patient reported nausea, vomiting, abdominal distension and constipation for 3 days. These symptoms started after dinner 3 days ago, and he had worsening abdominal pain without relief.

On presentation, the patient was afebrile, hemodynamically stable with body temperature of 36.4°C, heart rate of 84 per min and blood pressure of 117/83 mmHg. Blood work did not show leukocytosis (white blood cell count 9.01) or anemia (hemoglobin 16.9). Comprehensive metabolic panel was unremarkable (data not shown). Lactic acid was 2.5 mmol/L and was elevated above the normal limit (reference range 0.5–2.0 mmol/L).

At the time of exam, the patient was nauseous and actively vomiting with 10/10 diffuse abdominal pain not improved by intravenous morphine given in emergency department. Abdominal examination showed soft but distended abdomen and tenderness to palpation. The patient did not have obvious signs of peritonitis.

**Figure 1:**
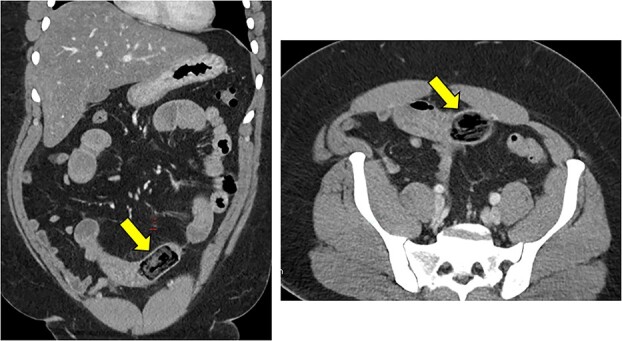
Dilated small bowel with a transition point in the distal small bowel with a rectangular object, which appeared to be an ingested foreign object.

Abdominal CT scan demonstrated small bowel obstruction with loops of small bowel in the central abdomen measuring up to 3.4 cm in diameter. A transition point was noted in the distal small bowel with a rectangular object, which appeared to be an ingested foreign object (Fig. 1).

Upon further questioning, the patient admitted to the consumption of suboxone (buprenorphine and naloxone) wrapped in a condom several years ago while he was incarcerated. He denied ever passing this object in stool. The patient also denied any recent ingestion of foreign bodies.

**Figure 2:**
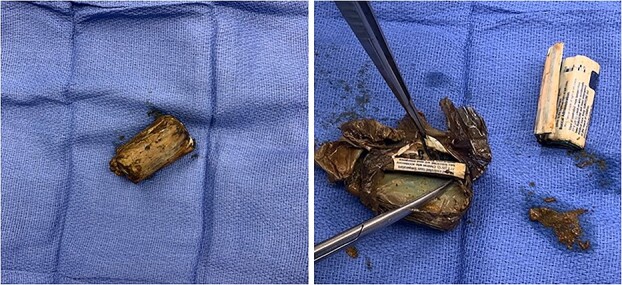
Back table inspection of the foreign body showing two rolled packets of suboxone sublingual film in two latex type balloons consistent with condoms.

The patient was taken urgently for exploratory laparotomy. The small bowel was eviscerated and an enterotomy was created proximal to the obstruction. The foreign body was removed. The enterotomy was resected and a side-to-side anastomosis was created using linear staplers. The abdomen was closed. The patient tolerated the procedure well. The foreign body was opened on the back table and was noted to be two rolled packets of suboxone sublingual film in two layers of blue-green latex type balloons, which is consistent with condoms (Fig. 2).

Postoperatively, the patient had delayed return of bowel function. Urine drug screening test resulted next day, and it was negative other than morphine that was administered in emergency department. On postoperative Day 7, the patient was able to tolerate regular diet and deemed stable to be discharged. The patient visited clinic on postoperative Day 14 for a follow-up. The patient was tolerating diet without any nausea, or emesis and demonstrated recovery from surgery.

## DISCUSSION

Body packing was first described in the medical literature in 1973 as a case report of a patient who swallowed a condom filled with hashish and developed small bowel obstruction 13 days later [5]. Body packing is not only associated with legal consequences but also with medical complications [6]. Body packers can suffer from acute drug toxicity from ingested drugs (heroin, cocaine or cannabis) or may require surgical intervention because of gastrointestinal obstruction or perforation [1, 6].

Small bowel obstruction is relatively infrequent complication of body packing. According to a retrospective study of 763 body packers in Spain, 16 patients (2.0%) suffered acute substance intoxication, 28 patients (3.6%) had small bowel obstruction and three patients (0.39%) developed bowel perforation [7]. Surgical intervention was required in only 28 (3.5%) patients. In another retrospective study of 45 patients in Iran, nine patients (20%) underwent surgery. Out of nine patients, eight patients (17.7%) underwent surgery because of toxicity with ingested drugs, and only one patient (2%) had gastrointestinal obstruction. The median number of packets ingested was significantly higher among patients who required surgical intervention (median 33.5, range 10–70), compared with those who were managed nonoperatively (median 2, range 1–16).

The reported incidence of small bowel obstruction is higher among body packers who ingests a higher number of drug packets. A 15-year retrospective study of body packers in New York City reported bowel obstruction occurred in nine (36%) out of 25 patients who required surgical intervention; 14 patients (56%) required surgery for sign of acute toxicity. The median number of ingested packets was 50 (range 1–120) among this study population [8]. Another study from the Caribbean Island of Curacao reported 57 (81.4%) out of 70 patients who required surgery had signs of either ileus or peritoneal irritation [9]. Although it is unclear how many of these patients had small bowel obstruction, the median number of ingested packets of this population was high with 30 (range 1–102), which likely contributed to a higher reported incidence of ileus or peritoneal irritation.

In the present case, the patient ingested a single suboxone-containing condom, which caused small bowel obstruction. The patient did not show any signs of acute drug toxicity, however had small obstruction with an obvious transition point demonstrated on CT scan with a foreign body concerning for illicit drug based on history. As a result, the patient required exploratory laparotomy and bowel resection. He claimed only remote history of body packing several years ago while being incarcerated and denied any recent ingestion of foreign objects. The literature shows that ingested drug packet can remain in the digestive tract for several days to even 4 weeks [2, 5, 9–11]. Although the exact timing of body packing by this patient cannot be proved, no previously reported case has such a longtime interval from the time of ingestion to medical presentation.

## Data Availability

The data underlying this article will be shared on reasonable request to the corresponding author.
